# Outcomes and Prognostic Factors of Posttraumatic Endophthalmitis: A Three-Year Retrospective Study

**DOI:** 10.1155/2021/5526998

**Published:** 2021-05-31

**Authors:** Jian Ma, Yinhui Yu, Yueyang Zhong, Xing Mao, Xiaoyun Fang

**Affiliations:** ^1^Department of Eye Center, the Second Affiliated Hospital, Zhejiang University School of Medicine, Hangzhou 310009, Zhejiang Province, China; ^2^Zhejiang Provincial Key Lab of Ophthalmology, Hangzhou 310009, Zhejiang Province, China

## Abstract

**Purpose:**

To describe the clinical features, management, and outcomes of patients with posttraumatic endophthalmitis (PTE) and to determine risk factors for poor visual prognosis.

**Methods:**

We retrospectively reviewed the medical records of 42 consecutive patients presenting with PTE who were treated at our institution between 2017 and 2019. Each patient's data, including demographic characteristics, ocular injury details, surgical records, patient outcomes, and laboratory results, were collected and analyzed. Multivariate analysis was conducted to determine the factors associated with poor visual outcomes.

**Results:**

In our series, male (*n* = 36, 85.7%) and patients below 60 years of age (20–40 years, 23.8%; 40–60 years, 57.14%) comprised most of the total cohort. On presentation, 39 (92.8%) of the 42 PTE patients presented best-corrected visual acuity (BCVA) worse than counting fingers. Pars plana vitrectomy (PPV) was performed in all the patients. 59.5% (*n* = 25) of the patients' BCVA improved after surgery and 33.3% (*n* = 14) achieved BCVA of 20/200 or better. The rate of evisceration was 7.1% (*n* = 3). Of the 42 specimens, the culture was positive in 10 (23.8%) eyes. By univariate analysis, factors including sex, occupation, systemic disease, source of trauma, lens injury, silicone oil tamponade, usage of intravitreal antibiotics, BCVA at presentation, and culture positive for any organism did not affect the final visual outcome. The features associated with poor BCVA (grouped as < 20/200 and ≥ 20/200) included older age (*P*=0.035), corneal-sclera wound (versus sclera wound) (*P*=0.047), retained intraocular foreign bodies (IOFBs) (*P*=0.006), treatment > 3 days (versus < 1 day) (*P*=0.033), and more times of surgeries (*P*=0.033).

**Conclusions:**

PTE is a severe complication of penetrating globe injuries associated with irreversible visual loss. Our results highlighted the importance of conducting early therapeutic PPV and IOFB removal to achieve better visual outcomes.

## 1. Introduction

The term “endophthalmitis” refers to infection of the vitreous, aqueous, or combination by bacteria or fungi [1]. Posttraumatic endophthalmitis (PTE) is an urgent devastating complication after penetrating ocular trauma, which may induce irreversible blindness in the infected eye [2, 3]. It is produced by microorganisms either gaining entry through an open wound or being introduced into the eye by contaminated injury-causing objects. In the reported series, PTE comprises approximately 25–30% of all endophthalmitis cases [2–4]. However, in some regions of China, PTE comprises almost 40–60% of all cases [1, 5], where industrial and agricultural injuries are much more common [6].

PTE usually has a poorer prognosis than other forms of endophthalmitis. The difficulties come from underlying eye trauma, poor visualization during surgery, and causation by more virulent germs [4, 7, 8]. PTE prognosis usually depends considerably on numerous factors, and it is critical to identify these features that influence the final vision and outcome regarding PTE. Although managing PTE poses a challenge to surgeons, recent advances in pathogen diagnosis, antibiotic treatment, and modern surgical techniques have remarkably improved the success rate [9–11]. However, numerous controversies still exist regarding the management modalities of PTE, necessitating an update of the prognostic factors associated with PTE for the appropriate treatment of the cases to improve visual outcomes.

Here, we retrospectively report our experience in managing consecutive patients with PTE for three years, describe the relationship between clinical presentation and visual outcome, identify risk factors affecting its prognosis, and further establish management modalities and other interventions. We expect that the risk factors identified in this study could help clinicians to establish a visual prognosis in PTE patients. Our information may potentially be critical in guiding clinicians in making optimal decisions regarding treatment strategies for this clinical challenge event.

## 2. Patients and Methods

### 2.1. Patients

This was a retrospective observational study at a large Eye Center, the Second Affiliated Hospital, Zhejiang University School of Medicine, Hangzhou, China, from January 2017 to December 2019. Inclusion criteria for PTE were the presence of symptoms (ocular pain, worsening vision, photophobia, and tearing) and deterioration in clinical signs (conjunctival hyperemia, purulent exudate at the site of injury, hypopyon, and vitreous opacification) following penetrating ocular trauma, which was defined as a full-thickness laceration of the eyewall in direct communication with the external environment with one entrance wound according to the International Statistical Classification of Diseases and Related Health Problems, 10th Revision codes [12].

### 2.2. Outcomes Analysis

The following data were extracted from medical records for each patient, including demographic data (gender, age, occupation, and systemic disease), ocular injury details (type of trauma, injury location, presence of hypopyon, and intraocular foreign bodies (IOFBs)), surgical records (modality and number of surgeries and elapsed time since endophthalmitis to surgery), patient outcome (preoperative and postoperative best-corrected visual acuity (BCVA), evisceration, or not), and laboratory results (culture and antibiotic sensitivities).

### 2.3. Surgical Technique

The decision for treatment was initiated as soon as possible without cultural confirmation. Pars plana vitrectomy (PPV) was recommended in any of the following situations: (1) extensive vitritis precluding visualization of the retina, (2) marked vitreous involvement by ultrasound B scan manifestation, (3) presence of IOFBs, and (4) virulent organism which is unresponsive to antibiotics or suspected fungal infections [13, 14]. Silicone oil or air tamponade was combined with PPV. All surgeries were performed by the same managing vitreoretinal surgeon (Jian Ma) using the Stellaris PC System (Bausch and Lomb, Rochester, New York, USA). Preoperatively, all endophthalmitis received intravitreal antibiotics (Vancomycin 1 mg/0.1 ml and Ceftazidime 2 mg/0.1 ml) according to our hospital's work modalities. At the end of the surgery, intravitreal antibiotics (Vancomycin 1 mg/0.1 ml and Ceftazidime 2 mg/0.1 ml) or antifungal agents (Amphotericin B 5ug/0.1 ml or Voriconazole 1 mg/0.1 ml) were only given in severe cases: (1) fast-growing inflammation, (2) incomplete PPV, and (3) suspected fungal infections. During hospitalization, systemic antibiotics or antifungal drugs were administered, and topical antibiotic and anti-inflammatory therapy (Tobradex, Pranoprofen) was maintained according to our experience or culture sensitivity reports if obtained. For microbiological analysis, 0.1 ml of aqueous humor and 0.5 ml of vitreous samples were manually aspirated before initiating the infusion. Smears were processed for Gram and Giemsa staining, bacterial and fungal cultures, and antibiotic sensitivities.

### 2.4. Statistical Analyses

Statistical analysis was performed using SPSS version 22.0.0 (Chicago, IL, USA). Frequencies and percentages were reported for all variables. Data were assessed for normality using the Shapiro–Wilk test. Means were compared using *t*-tests for normally distributed variables or the Mann–Whitney *U* test for nonnormally distributed variables. Categorical variables were analyzed using the chi-square test or Fisher's exact test. *P* < 0.05 was considered statistically significant.

## 3. Results

We identified 42 cases that exhibited PTE during the three years at our institution (36 males and 6 females). The mean age was 44.43 ± 14.68 years (range: 7 to 73 years). Notably, patients aged from 20 to 40 years comprised 23.8% of the total cohort, and those aged from 40 to 60 years comprised 57.14% of the total cohort. Only six patients had systemic disease, with the diagnoses being hypertension (*n* = 4; 9.5%), diabetes (*n* = 1; 2.4%), and Parkinson's disease (*n* = 1; 2.4%). Industrial worker (*n* = 23; 54.8%) was the most frequently identified occupation, followed by farmer (*n* = 13; 30.9%) and others (*n* = 6; 14.3%). The average follow-up period was 15.07 ± 4.63 months.  Table 1 shows the demographic characteristics of the patients.

The wounds affected cornea (*n* = 18; 42.8%), sclera (*n* = 13; 30.9%), and corneal-sclera (*n* = 11; 26.2%). Type of injury included sharp (*n* = 32; 76.2%) and blunt (*n* = 10; 23.8%). The most common etiology of sharp injuries was iron (*n* = 21; 50%), followed by steel (*n* = 8; 19%). In blunt injuries, stone (*n* = 4; 9.5%) and hard objects (*n* = 3; 7.1%) were most common. Clinical examinations showed hypopyon in 29 (69%) patients at the presentation. Other combined injuries included lens capsule rupture with the formation of traumatic cataracts (*n* = 31; 73.8%) and retained IOFBs (*n* = 16; 38.1%).  Table 2 presents a detailed description of the mechanisms of injury.

The average treatment delay was 3.67 ± 2.74 days (range: 1 to 14 days). Among them, surgical treatment was within 1 day of injury (*n* = 14; 33.3%), between 1 and 3 days (*n* = 18; 42.8%), and more than 3 days (*n* = 10; 23.8%). Treatment modalities included PPV (*n* = 42; 100%), combined cataract removal (*n* = 31; 73.8%), primary IOL implantation (*n* = 6, 14.3%), combined silicone oil (*n* = 23; 54.8%), and air tamponade (*n* = 19, 45.2%). In severe cases, intravitreal drugs (*n* = 7; 16.7%) were given. The mean number of surgical procedures performed was 1.95 ± 0.87; twenty patients required two procedures (47.6%), seven patients required three procedures (16.7%), and two patients required four procedures (4.8%).  Table 3 depicts the distribution of the various treatment modalities of the patients.

All PTE patients in this study population showed decreased vision. On presentation, only 1 (2.4%) patient had a BCVA of 20/200 or better, and 39 (92.8%) patients showed BCVA worse than counting fingers. Generally, more than half (*n* = 25; 59.5%) of the cases achieved final visual improvement after surgery, which was 20/200 or better (*n* = 14; 33.3%), less than 20/200 (*n* = 3; 7.1%), counting fingers (*n* = 4; 9.5%), hand motions (*n* = 11; 26.2%), and light perception (*n* = 7; 16.7%). Also, 3 (7.1%) patients were left with no light perception and underwent evisceration.  Table 4 shows the detailed distribution of BCVA at presentation and the final follow-up in study subjects.

Furthermore, of the 42 specimens, the culture was positive in only 10 (23.8%) eyes. Of the positive, Gram-positive (*n* = 6; 14.3%) and Gram-negative (*n* = 4; 9.5%) germs were isolated. For Gram-positive germs, *Staphylococcus epidermidis* (*n* = 4, 9.5%) was the most frequent, followed by *Staphylococcus saprophyticus* (*n* = 2, 4.8%). Gram-negative germs were isolated in *Bacillus cereus* (*n* = 1, 2.4%), *Enterobacter cloacae* (*n* = 1; 2.4%), *Lactococcus lactis* (*n* = 1; 2.4%), and multiple organisms (*n* = 1, 2.4%). Notably, in the present study, all *Staphylococcus epidermidis* infections were in cases with IOFBs. Also, of the three cases that underwent evisceration, two were Gram-negative bacilli infections.  Table 5 depicts the distribution of the pathogens detected.

Table 6 shows the results of the univariate analysis for determining the independent risk factors for poor visual prognosis. For ease of presentation, the final BCVA was divided into two groups, namely, <20/200 and ≥ 20/200. Statistically, multiple factors, including sex, occupation, systemic disease, source of trauma, lens injury, silicone oil tamponade, usage of intravitreal antibiotics, BCVA at presentation, and culture positive for any organism, did not affect the final visual outcome. The features associated with BCVA <20/200 included older age (*P*=0.035), corneal-sclera wound (versus sclera wound) (*P*=0.047), retained IOFBs (*P*=0.006), treatment > 3 days (versus < 1 d) (*P*=0.033), and more times of surgeries (*P*=0.033).

## 4. Discussion

Posttraumatic endophthalmitis is an uncommon but severe complication of penetrating globe injuries associated with irreversible vision loss. Due to the large population and poor protection awareness for ocular trauma, PTE is common in our country [7, 14, 15]. PTE prognosis is affected by various factors, and it is valuable to analyze this condition separately. In this study, we retrospectively reviewed our practice for treating PTE cases and analyzed the controversial issues that may affect their visual outcome. Overall, we identified five clinically relevant factors associated with worse visual outcomes in our series.

Unsurprisingly, most (85.7%) of the PTE patients were males, and industrial workers comprised 54.8% of all patients in our series. In particular, young workers (20 to 50 years) comprised 82.6% (*n* = 19) of the total workers. This is mostly because, in Eastern China, a major part of the population is engaged in industry, and young men tend to suffer more from trauma in their high-risk workplace. We found an association between older age and poor BCVA (*P*=0.035). We found that the age in the group with final BCVA ≥ 20/200 was 37.29 ± 15.90 years compared with age in the group with final BCVA < 20/200 of 48.00 ± 12.86 years. This followed a previous study, which showed an increased risk of endophthalmitis in patients above 50 years of age [2], and the EVS study, which reported that older age was an independent risk factor for decreased BCVA after endophthalmitis [16]. Additionally, we found a delayed time lapse in endophthalmitis to repair in the greater than 48 years of the age group of 4.23 days (versus < 48 years, 3.05 days, *P* > 0.05). We speculated that a lack of awareness or access to prompt ophthalmological emergency care contributed to their poor prognoses.

In our series, we revealed that patients with corneal-sclera (versus sclera) wounds carried worse visual prognosis. Final BCVA ≥ 20/200 was achieved in 61.5% of patients with sclera wounds (versus corneal-sclera injury, 18.2%). The univariate analysis revealed that the presence of a corneal-sclera wound was an independent risk factor for poor vision (*P*=0.047). A previous study has also identified that a corneal-scleral wound (versus corneal wound) was associated with poor visual prognosis, even in the absence of endophthalmitis [2], which was confirmed in our study. We speculated that lesions in the anterior segment of the eye are usually associated with concomitant injuries of corneal astigmatism and delayed healing of the corneal limbus [17], which may prevent satisfactory recovery of ultimate BCVA. Moreover, posterior scleral lacerations are commonly associated with vitreous and uveal prolapse, which has been validated to produce a barrier against microorganisms to prevent continued inflammation. However, further studies are needed to investigate whether the previous findings are consistent and sustained.

The presence of IOFBs also increased the risk of poor visual outcomes. Of the 16 cases with IOFBs, almost all (*n* = 15; 93.7%) only attained a final BCVA < 20/200. The analysis demonstrated that retained IOFBs were an independent risk factor for poor vision compared to those without (*P*=0.006). Moreover, three cases that underwent evisceration had IOFBs. In our previous study, we showed that the presence of IOFBs contributed to 21.4% of the rate of endophthalmitis [18]. IOFBs affect PTE prognosis in the following ways: (1) anatomical structure damage like PVR and retinal tear produced by IOFBs, (2) inflammatory chemistry of the IOFBs, and (3) carrier to deliver infectious agents [19, 20]. Thus, we can conclude that the presence of IOFBs progresses the existing endophthalmitis and further adds difficulties to vitreous surgery, thereby contributing to worsening the prognosis. Based on our experience, we advise the ophthalmologist to be aware of retained IOFBs, and prompt evaluation and surgical removal are therefore recommended.

According to the statistics, surgical treatment was performed for 14 (33.3%) of the patients within 1 day, and 8 (57.1%) of them recovered a BCVA of 20/200 or better. In contrast, 9 (90%) of 10 patients, in which treatment was initiated after 3 days due to a delay of seeking medical care, ended up with a BCVA of 20/200 or worse (*P*=0.033). We noted a protective effect against poor vision if the primary treatment was performed within 1 day of the presentation of endophthalmitis. This result was also verified by previous retrospective studies, which reported that poor visual recovery from endophthalmitis was associated with a delay in diagnosis and treatment [21]. We, therefore, advocated prompt intervention of PTE preferable within 1-day manifestation, which may allow better visual recovery.

In the case of PTE, appropriate therapy must be adopted to control infections and minimize ocular morbidity. In our cohort, treatment modalities included PPV (*n* = 42; 100%), combined cataract removal (*n* = 31; 73.8%), primary IOL implantation (*n* = 6, 14.3%), combined silicone oil (*n* = 23; 54.8%), and air tamponade (*n* = 19, 45.2%). Statistically, the treatments did not influence the final visual prognosis. However, by adopting PPV in all patients, the evisceration rate of the study population was only 3 (7.1%). It has been verified by studies that vitrectomy can (1) clear the media by removing vitreous membranes and debris that may cause retinal detachment via traction effects; (2) remove infectious inoculum, toxins, and clear inflammatory cells; and (3) reduce microbiologic load within the eye and allow for better diffusion of intravitreal antibiotics [11, 22]. Overall, we recommended early PPV in PTE cases. However, we found that more surgical procedures were associated with worse visual prognosis, probably because the patients who underwent multiple surgeries were mostly severe cases in which the inflammation was uncontrolled or with retinal detachment, the inflamed retina can be fragile and prone to tears, and continuing surgery in these situations is risky. Moreover, multiple surgeries may contribute to a higher risk of proliferative vitreoretinopathy (PVR) due to the increased breach of the blood-retinal barrier, which exacerbated retinal necrosis and delayed healing with very poor BCVA [23, 24].

Overall, the visual outcome in traumatized eyes with endophthalmitis is extremely poor. Among the 42 cases, the final BCVA was 20/200 or better in only 33.3% of patients. It may be due to the nature of trauma, which posed a major effect on visual prognosis. However, we did not identify any evidence that cases with better BCVA at presentation had a better final visual outcome (*P*=0.102). These results are not in line with the results of our previous retrospective study of IOFBs in those patients with more favorable initial BCVA tending to achieve better final visual outcomes [18]. Moreover, the EVS data also found that patients presenting with BCVA of LP are twice as likely to have decreased visual outcomes compared to those presenting with BCVA > LP [16]. We considered that visual acuity may not be the sole variants that could influence the final visual prognosis. We think that more studies are needed to determine any associations between these factors.

Identification of causative pathogens is an important step in managing infective endophthalmitis. Of the 42 specimens, the culture was positive in only 10 (23.8%) eyes, which agreed with the reported rate of positivity in previous studies [25,26]. The presence of positive or negative cultures in cases of PTE did not affect the visual outcome (*P*=0.451). Gram-positive organisms were the predominant isolates in PTE cases, in which 2 (33.3%) of the six cases of *Staphylococcus epidermis* infections achieved a final BCVA of 20/200 or better. However, in Gram-negative *Bacillus* infections, 2 (50%) ended up with evisceration, and the other two cases achieved only a final BCVA of LP. In three cases that underwent evisceration, two were affected by *Bacillus cereus*, either as a sole causative agent or multiple infections; the results demonstrated that this organism was associated with more virulent and severe infections, which had a high risk of progressing to the final BCVA of NL [27].

Limitations of this study included its retrospective nature, relatively small number of patients, the inclusion of a single hospital's experience, and the large variability of presentations, making the cases difficult to standardize. As a hospital-based study, selection bias might exist in our population of referred patients, which may represent more severe cases. Additionally, more information, including the degree of wound contamination, ocular trauma scoring, and primary repair before treatment, was unavailable. Nonetheless, to our knowledge, this study strengthened the risk factors that may explain the differences in the visual prognoses of PTE patients.

## 5. Conclusions

Our study showed that PTE patients with older age, corneal-sclera wounds, retained IOFBs, delayed treatment, and more times of surgeries had a higher risk of the poor final vision. Compared to postsurgery endophthalmitis, traumatic endophthalmitis is generally more severe and is associated with more complicated surgical procedures, including repeated PPV or complicated surgeries. We recommended conducting early therapeutic PPV and IOFB removal to attain a better visual outcome. However, prevention of ocular trauma is still critical.

## Figures and Tables

**Table 1:** Demographic characteristics of patients.

Variables	Number (%) or mean (SD)
Age	44.43 (14.68)

Sex	
Male	36 (85.7)
Female	6 (14.3)

Systemic disease	
Hypertension	4 (9.5)
Diabetes	1 (2.4)
Parkinson	1 (2.4)

Occupation	
Industrial worker	23 (54.8)
Farmer	13 (30.9)
Others	6 (14.3)
Follow-up	15.07 (4.63)

**Table 2:** Mechanisms of injury.

Variables	Number (%)
Site of laceration	
Cornea	18 (42.8)
Sclera	13 (30.9)
Corneal-sclera	11 (26.2)

Type of injury	
Sharp	32 (76.2)
Blunt	10 (23.8)

Sharp	
Iron	21 (50)
Steel	8 (19)
Electric drill	1 (2.4)
Pencil	1 (2.4)
Bamboo	1 (2.4)

Blunt	
Stone	4 (9.5)
Hard object	3 (7.1)
Air nail gun	2 (4.8)
Chinese chestnut	1 (2.4)

Hypopyon	
Yes	29 (69)
No	13 (30.9)

Traumatic cataract	
Yes	31 (73.8)
No	11 (26.2)

Retained IOFBs	
Yes	16 (38.1)
No	26 (61.9)

**Table 3:** Treatment modalities of patients.

Variables	Number (%) or mean (SD)
Interval to first surgical procedure	3.67 (2.74)
<1 d	14 (33.3)
1–3 d	18 (42.8)
>3 d	10 (23.8)

Treatment modality	
PPV	42 (100)
Cataract removal	31 (73.8)
Primary IOL implantation	6 (14.3)
Silicone oil tamponade	23 (54.8)
Air tamponade	19 (45.2)
Intravitreal drugs	7 (16.7%)

Number of surgeries	1.95 (0.87)
1	13 (30.9)
2	20 (47.6)
3	7 (16.7)
4	2 (4.8)

**Table 4:** Visual outcomes of patients.

Variables	Preoperative BCVA, *n* (%)	Postoperative BCVA, *n* (%)
≥20/200	1 (2.4)	14 (33.3)
<20/200	2 (4.8)	3 (7.1)
CF	5 (11.9)	4 (9.5)
HM	17 (40.5)	11 (26.2)
LP	15 (35.7)	7 (16.7)
NLP	2 (4.8)	NA
Evisceration	NA	3 (7.1)
Total	42 (100)	42 (100)

Abbreviations: BCVA, best-corrected visual acuity; CF, counting fingers; HM, hand motions; LP, light perception; NLP, no light perception; NA, not available.

**Table 5:** Pathogens isolated in the patients.

Variables	Number (%)
Gram-positive germs	
*Staphylococcus epidermidis*	4 (9.5)
*Staphylococcus saprophyticus*	2 (4.8)

Gram-negative germs	
*Bacillus cereus*	1 (2.4)
*Enterobacter cloacae*	1 (2.4)
*Lactococcus lactis*	1 (2.4)
*Pseudomonas aeruginosa* + *Bacillus cereus*	1 (2.4)

**Table 6:** Risk factors for poor visual outcomes.

Variables	Final BCVA <20/200, *n* (%)	Final BCVA ≥20/200, *n* (%)	Or (95% CI)^a^	*P* value^b^
Age (by year)	48.00 ± 12.86	37.29 ± 15.90	1.055 (1.004, 1.108)^c^	0.035^d^

Sex				1.000
Male	24	12	1.000 (0.160, 6.255)	
Female	4	2	1	

Occupation				0.225
Farmer	11	2	1	
Worker	14	9	0.283 (0.050, 1.585)	0.259
Others	3	3	0.182 (0.020, 1.638)	0.262

Systemic disease				0.083
Yes	6	0	1.636 (1.261, 2.123)	
No	22	14	1	

Methods				0.094
Iron	16	5	1	
Steel	5	7	0.223 (0.049, 1.026)	0.067
Others	7	2	1.094 (0.169, 7.061)	1.000

Sharp materials				0.451
Yes	20	12	2.400 (0.435, 13.227)	
No	8	2	1	

Injured part				0.034
Combined	9	2	1	
Cornea	14	4	0.778 (0.117, 5.162)	1.000
Sclera	5	8	0.139 (0.021, 0.925)	0.047

Preoperative VA				1.000
≥CF	11	3	0.797 (0.161, 3.954)	
<CF	23	5	1	

Cataract				0.321^e^
Yes	22	9	2.037 (0.493, 8.408)	
No	6	5	1	

Intraocular foreign body				0.006
Yes	15	1	15.000 (1.721, 130.759)	
No	13	13	1	

Phase I treatment				0.107^e^
Yes	18	5	3.240 (0.849, 12.360)	
No	10	9	1	

Use of silicone oil				0.826^e^
Yes	15	8	0.865 (0.237, 3.153)	
No	13	6	1	

Intraocular injection				1.000
Yes	5	2	1.304 (0.219, 7.751)	
No	23	12	1	

Bacterial culture				0.451
Positive	8	2	2.400 (0.435, 13.227)	
Negative	20	12	1	

Hypopyon				0.723
Yes	20	11	0.682 (0.150, 3.109)	
No	8	3	1	

Time of operation				0.043
0–24 h	9	1	1	
24–72 h	6	8	0.083 (0.008, 0.849)	0.033
>72 h	13	5	0.289 (0.029, 2.908)	0.375

Abbreviations: VA, visual acuity; OR, odds ratio; 95% CI, 95% confidence interval. (a) Logistic regression analysis. (b) Fisher's exact test. (c) Reference age has OR = 1. (d) Student t-test. (e) Chi-square analysis.

## Data Availability

The datasets used or analyzed during the current study are available from the corresponding author upon reasonable request.

## Abbreviations

PTE: Posttraumatic endophthalmitis
PPV: Pars plana vitrectomy
IOFBs: Intraocular foreign bodies
PVR: Proliferative vitreoretinopathy
BCVA: Best-corrected visual acuity
CF: Counting fingers
HM: Hand motions
LP: Light perception
NLP: No light perception
NA: Not available.
